# Support mechanical response analysis and surrounding rock pressure calculation method for a shallow buried super large section tunnel in weak surrounding rock

**DOI:** 10.1038/s41598-024-64522-6

**Published:** 2024-06-12

**Authors:** Haixiang Lai, Xiuying Wang, Zhongsheng Tan, Jinpeng Zhao, Xiabing Liu

**Affiliations:** 1 Key Laboratory of Urban Underground Engineering of Ministry of Education, Beijing Jiaotong University, Beijing, 100044 China; 2 School of Civil Engineering, Beijing Jiaotong University, Beijing, 100044 China; 3 State Key Laboratory of Hydroscience and Engineering, Tsinghua University, Beijing, 100084 China; 4 Guangdong Provincial Key Laboratory of Tunnel Safety and Emergency Support Technology and Equipment, Guangzhou, 510420 Guangdong China

**Keywords:** Super-large-section tunnel, Weak surrounding rock, Shallow-buried, Analysis of deformation and mechanical response, Surrounding rock pressure calculation method, Civil engineering, Petrology

## Abstract

At present, China's demand for high-speed railway construction is constantly increasing, and the construction of Multi line high-speed railway tunnels has been put on the agenda. The design and construction issues of super-large-sections tunnels urgently need to be addressed. The Xiabei mountain No. 1 and No. 2 tunnels in the Hangzhou-Taizhou Railway are typical shallow-buried super-large-section-tunnels in weak surrounding rock, and their design and construction issues are representative. Eleven monitoring sections were set up in the tunnel, including tunnel deformation, surrounding rock, shotcrete, steel frames, bolts and temporary support mechanical responses. Taking the monitoring data of the most typical cross-section as an example, the mechanical response of the support structure of a shallow-buried super-large-section tunnel was analyzed in detail. Based on previous research results, this paper discusses and summarizes the common construction problems of this type of tunnel, and puts forward corresponding suggestions. The existing formula for calculating surrounding rock pressure has poor applicability to super-large-section tunnels constructed by step excavation, resulting in conservative support parameters. Therefore, based on the monitoring values of surrounding rock pressure at 10 monitoring sections in Xiabei mountain No. 1 and No. 2 tunnels, empirical parameters reflecting the impact of step excavation were summarized. Based on the Wang formula and combined with the step excavation empirical parameters, an empirical formula for the surrounding rock pressure of shallow-buried super-large-section tunnels considering step excavation was constructed. The calculated results are in good agreement with the on-site monitoring data. This study can provide a good reference for similar projects.

## Introduction

With the increasing demand for transportation among the Chinese people, the construction demand for super-large-section multi lane tunnels has also increased^1–3^. Typical super-large-section traffic tunnels include: Gongbei tunnel, Longding tunnel and so on^4,5^. These types of tunnels are often constructed using a step excavation method, during which the surrounding rock and support structures are constantly disturbed with the segmented excavation, resulting in changes in the surrounding rock pressure. Therefore, the construction mechanical response becomes more complex^6–9^. Exploring the construction mechanical response and surrounding rock pressure variation law of this type of tunnel can effectively guide the design and construction, which is of great significance for the construction of this type of tunnel.

The study of the above issues through model test, numerical simulation and analytical solution methods has the characteristics of flexibility and simplicity, and many scholars have conducted in-depth discussions^10,11^. He et al.^12^ studied the application of negative Poisson's ratio bolts combined with excavation compensation method in super large span tunnels based on model experiments. Li et al.^13,14^ used large-scale geo-mechanical model tests to study the failure characteristics and dynamic pressure arch phenomenon of large-section tunnels at different burial depths. Wang et al.^4^ studied the stress and deformation characteristics of composite arches and steel frames in large-section tunnels using model testing methods. Zhang et al.^15^ conducted model test base on the Xinwu super-large-section tunnel, and the deformation of surrounding rock, cracking of lining, stress, and bearing capacity of lining in the experiment were analyzed and discussed. Zhang et al. used the mini multipoint extensometer as the monitoring equipment for the geomechanical model test of the large-span caverns to analyze the mechanical response of the large-span caverns^16–18^. Luo et al.^19^, Luo et al.^20^ and Shi et al.^21^ conducted a study on the construction methods, design, and surrounding rock stability of large-span tunnels using numerical simulation methods combined with on-site monitoring data. Some researchers have also used numerical simulation methods to study the excavation damage zone and surrounding rock stability of super large cross-section tunnels^9,22,23^. Lin et al.^24^ and Luo et al.^6^ conducted enhanced design work for large span tunnels based on analytical solutions and numerical simulation methods. However, the above methods ignore the uncertainty of on-site construction and cannot reflect the actual construction situation. At the same time, the research object is a deep buried large cross-section tunnel, and the construction difficulty is significantly lower than that of the Xiabei mountain Tunnel, so the reference value of the research results is limited.

However, numerical simulation and model testing are difficult to fully reproduce the on-site situation, and the actual on-site monitoring results are still crucial. Numerous scholars have used on-site monitoring methods to investigate the mechanical response of super-large-section tunnel^25,26^. Zhang et al.^27^ used on-site monitoring methods to study the construction mechanical response and deformation characteristics of large-span and small spacing tunnels. Guo et al.^28^ used on-site monitoring methods to study the application of negative Poisson's ratio bolts in super large span tunnels. Li and Zhao^29^ analyzed the mechanical response of loess tunnels using step excavation method based on on-site monitoring data. Li et al.^30^ discussed the failure mechanism of super-large-section tunnels in near horizontal strata based on monitoring data. Zhang et al.^31^ analyzed the surface deformation of a super-large-section tunnel constructed by the freezing method based on on-site monitoring data. Li et al.^13^ investigated the distribution and variation patterns of surrounding rock pressure in large-section tunnels based on on-site monitoring data. Luo et al.^32^ monitored and analyzed the support mechanical response of the Badaling super-large-section underground station. Previous studies have analyzed and summarized the actual construction mechanical response of large span tunnels, but the excavation methods are different and the tunnel span is smaller than that of the Xiabei mountain Tunnel. Its reference value is still limited, and it cannot meet the design and construction needs of shallow buried soft rock super large section tunnels such as the Xiabei mountain Tunnel.

The rock pressure law obtained solely from model tests, numerical simulations, or monitoring results still cannot meet the requirements of construction and design. The calculation formula for surrounding rock pressure obtained through theoretical or empirical methods is more practical. However, the applicability of existing standardized methods for calculating rock pressure in super large section tunnels is poor, and the development of rock pressure calculation formulas suitable for such tunnels has attracted the attention of many scholars. Lei et al.^33^ derived a formula for calculating surrounding rock pressure based on the Terzaghi theory combined with nonlinear yield criteria. Luo et al.^34^ studied the geological response curve of super-large-section tunnels in strain softened rock masses using analytical methods. Gao et al.^35^ discussed the formula for calculating the surrounding rock pressure of super-large-span tunnels. Luo et al.^36,37^ derived a formula for calculating the surrounding rock pressure of large-span tunnels considering step excavation, enriching relevant research results. The above research proposes excellent methods for calculating surrounding rock pressure, but neglects a series of unfavorable factors caused by size effects and step excavation. The applicability of shallow buried soft rock super large section tunnels such as the Xiabei mountain tunnel needs to be verified.

Based on the research results, it is known that the support mechanical response and surrounding rock pressure of super-large-section tunnels are more complex than conventional tunnels. If constructed in weak and fractured strata with shallow overburden, the above problems will worsen. Therefore, this article conducts the following research work based on the Xiabei mountain tunnel under the Hangzhou-Taizhou high-railway, which is a shallow-buried super-large-section tunnel in weak surrounding rock. Firstly, based on on-site monitoring of tunnel deformation, surrounding rock pressure, shotcrete mechanical response, steel frame mechanical response, temporary support mechanical response, and bolts axial force, the deformation and mechanical characteristics are analyzed. Through discussion with similar engineering monitoring results, the mechanical response of the support structure of this type of tunnel is summarized. Secondly, based on the formula for calculating the safety factor of shotcrete in the TB 10003-2016. 2016^38^. Code for design of railway tunnel, the safety and economic issues of the support structure design of the tunnel are determined. Finally, based on the surrounding rock pressure monitoring data of 10 sections in Xiabei mountain tunnel, empirical parameters considering step excavation were constructed. Base on the step excavation empirical parameters, the calculation formula for the surrounding rock pressure of shallow-buried super-large-section tunnels was derived. For super-large-section tunnels excavated by step excavation, compared to traditional formulas, this formula calculates accurately and can obtain the surrounding rock pressure values for each pilot tunnel. This article explores the construction mechanical response and rock pressure variation law of shallow buried super large span tunnels in weak surrounding rock, and the research results have good reference value for similar projects.

## Engineering background

### Geological overview

**Figure 1:**
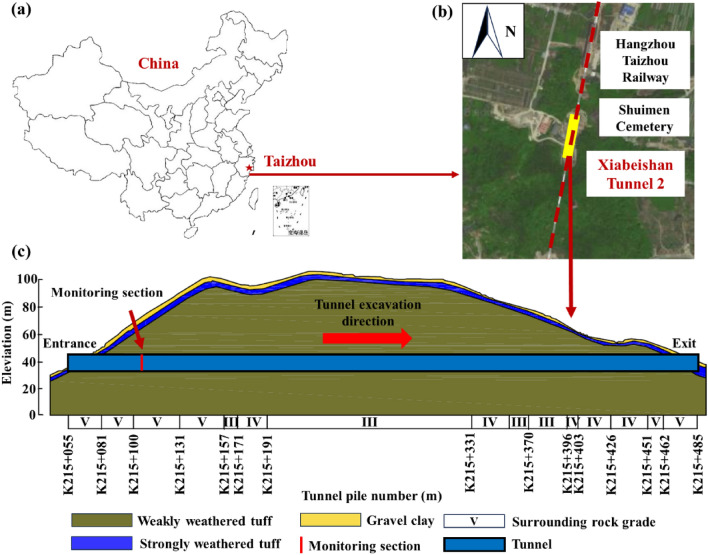
Geological environment of Xiabei mountain No. 2 tunnel: (**a**) The location of tunnel; (**b**) Surrounding environment of tunnel; (**c**) Geological planning map.

The Xiabei mountain Tunnel of the Hangzhou-Taizhou high speed railway is located in Taizhou, Zhejiang Province, China (Fig. 1a). The Xiabei mountain tunnel No. 2 passes through the hilly terrain of eastern Zhejiang. The terrain of the tunnel site is undulating, with a slope of 25°–45°, and the mountain is covered by the Quaternary soil layer (Fig. 1b). The depth range of the tunnel is only 6–35 m, and the east side of the tunnel is adjacent to the cemetery and high-voltage power tower, which requires relatively high deformation control of the surrounding rock. The longitudinal section diagram of the tunnel is shown in Fig. 1c, the surface is covered with a layer of silty clay with a thickness of 0.5–4 m. The excavation area of the tunnel is completely weathered and strongly weathered tuff. There are clay interlayers with a thickness of 10–50 mm in joints. Based on the BQ system, it is determined that the entrance section is mainly composed of class V surrounding rock, while the tunnel section is mainly composed of class III and IV surrounding rock. At the same time, due to the need to reserve high-speed railway lines, the tunnel is designed as a four line type. When crossing the Xiabei mountain, the thickness range of the soil cover is only 6–35 m. The east side of the tunnel is adjacent to the cemetery and high-voltage power tower, and the requirements for controlling the deformation of the surrounding rock are relatively high.

### Design and construction overview

**Figure 2:**
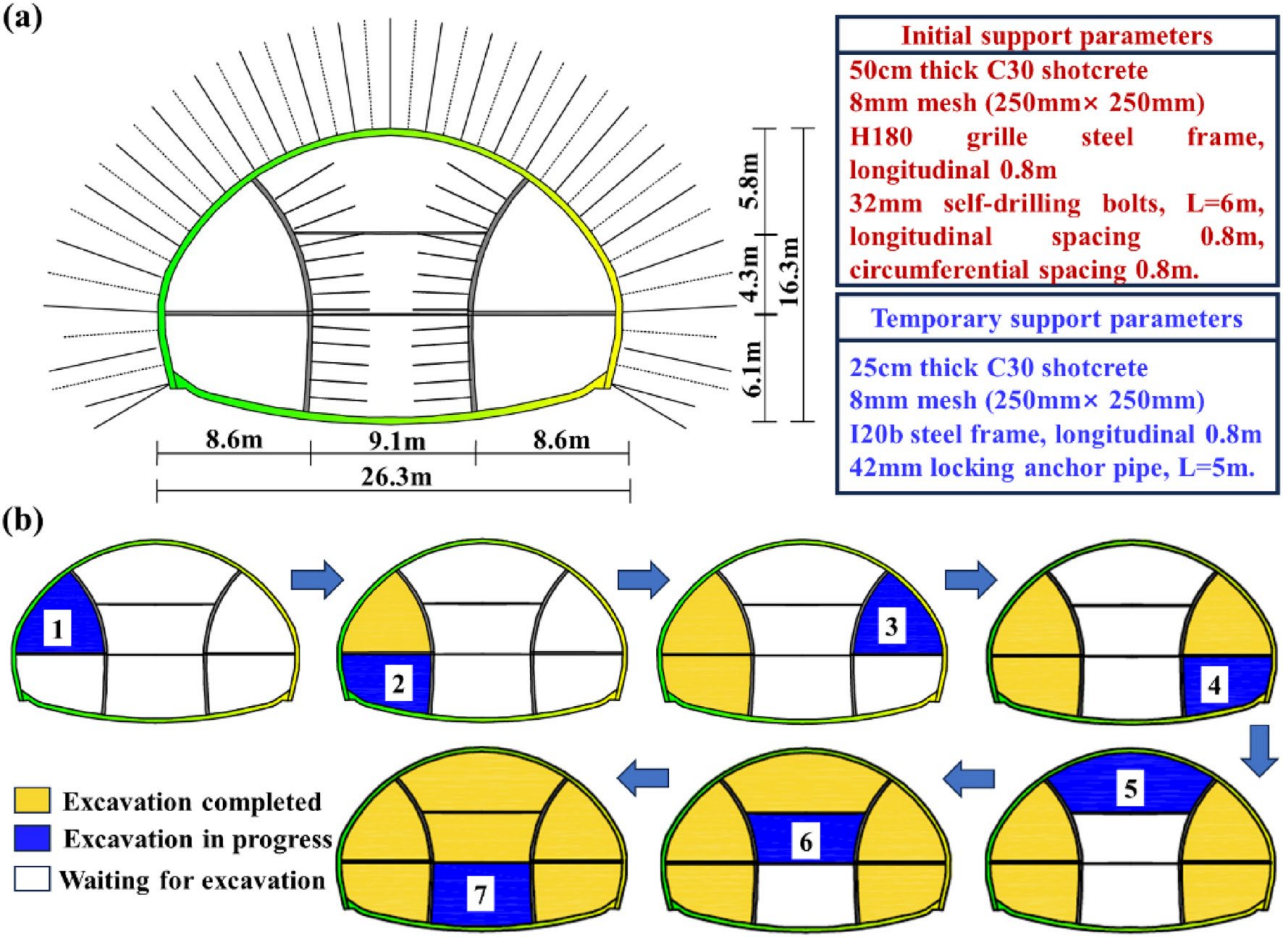
Construction scheme of Xiabei mountain No. 2 tunnel: (**a**) Parameters of primary supporting structures; (**b**) Excavation method.

The tunnel is constructed using the drilling and blasting method, with an excavation span and excavation area of 26.3 m and 361.4 m^2^, respectively. Due to the large excavation area, special tunnel cross-sectional shape, and short distance of tunnels, using shield TBM or TBM methods for excavation cannot meet the requirements of excavation area and tunnel cross-sectional shape, and the economy is poor. The drilling and blasting method has advantages such as flexibility, economy, and simple operation when excavating such tunnels, so it is appropriate for the Xiabei mountain No. 2 tunnel. However, blasting can disturb the surrounding rock, which can make it more unstable, this poses challenges to construction technology and design requirements. Based on TB 10003-2016. 2016. Code for design of railway tunnel.^38^ and Zhao et al.^39^ determined that the burial depth of the Xiabei mountain tunnel is shallow. Due to the excavation span reaching 26.3 m, in order to reduce the disturbance of excavation on the surrounding rock, choose the double-side-wall pilot tunnel method for construction. In the on-site study, a total of 10 cross-sections were monitored. Considering that shallow-buried super-large-section tunnels with class V rock mass are more rare and have research value, the DK215 + 105 section with the most unfavorable rock mass strength in the tunnel was selected as the key research object to study the deformation and mechanical characteristics of the primary support system during the construction period. Due to the lack of similar engineering experience during construction, the common types of tunnels are Class III and Class IV deep-buried super-large-section tunnels with surrounding rocks, and the current tunnel design specifications do not provide support design guidance for four line tunnels^40–43^. Therefore, when designing the support of this tunnel, only a small number of engineering cases can be relied on for analogy design, and the design parameters are relatively conservative. The specific primary support parameters for class V surrounding rock are shown in Fig. 2a.

The specific construction process includes 8 steps, and the excavation sequence of the double-side-wall pilot tunnel method is shown in Fig. 2b:

1. Excavation of left and right pilot tunnels: (1) Excavate the upper steps of the left pilot tunnel, and the excavation cycle footage is a steel frame (1.2 m). Apply the primary shotcrete (including peripheral shotcrete and temporary shotcrete) around this part. Construction Φ 32 self-drilling bolts with a length of 6 m, a circumferential spacing of 0.8 m, and a longitudinal spacing of 0.8 m. Both rock bolts and cables require drilling, and drilling in soft rock can easily lead to collapse, making it very difficult to install bolts or cables. Therefore, the project adopts self advancing bolts to avoid various problems that may occur during drilling. Erect the steel frame at the arch section, and set up feet-lock bolt at an inclination angle of 30°, close to the edges of both sides of the steel frame at a height of about 30 cm above the arch foot. The feet-lock bolt are firmly welded to the steel frame, and the excavation is 2.4 m. (2) The construction method for the lower step of the left pilot tunnel is the same as that for the upper step. (3) The construction method for the right and left pilot tunnels is the same, with a construction spacing of 2.4 m between each part.
2. To reduce the impact of excavation blasting vibration, one to four inner side walls and horizontal temporary steel frames will be erected approximately 10 m away from the front face of the tunnel.
3. Excavation of part 5 of the main tunnel: Excavation is divided into two parts: left and right. After excavation, primary support and temporary support are provided, with a distance of 2.4 m between the left and right parts. Finally, the primary supports will be closed.
4. Demolition of temporary supports: After excavation of part 5, in order to minimize the transmission of disturbing from part 6 and 7 to the primary support of the closed primary support, the temporary steel frames in parts 1–5 are removed.
5. Excavate parts 6 and 7 and application of shotcrete to the design thickness.
6. After the primary support closure, steel fiber reinforced concrete was sprayed for reinforcement, lagging behind the primary support closure by 2.4 m.
7. Spray a 1 cm thick cement mortar protective layer.
8. Construction the secondary lining concrete.

The physical and mechanical parameters of the monitoring section surrounding rock are shown in Table 1.

**Table 1:** Physical and mechanical parameters of the rock mass in the monitoring section.

Stratum	Elastic modulus (GPa)	Poisson's ratio	Cohesion (kPa)	Internal friction angle (°)	Density (kN m^−3^)
Residual soil	0.07	0.42	31	25.5	19.5
Tuff	0.52	0.26	113	27.3	25.7

## Analysis of surrounding rock and support structures on-site monitoring

### Monitoring scheme

**Figure 3:**
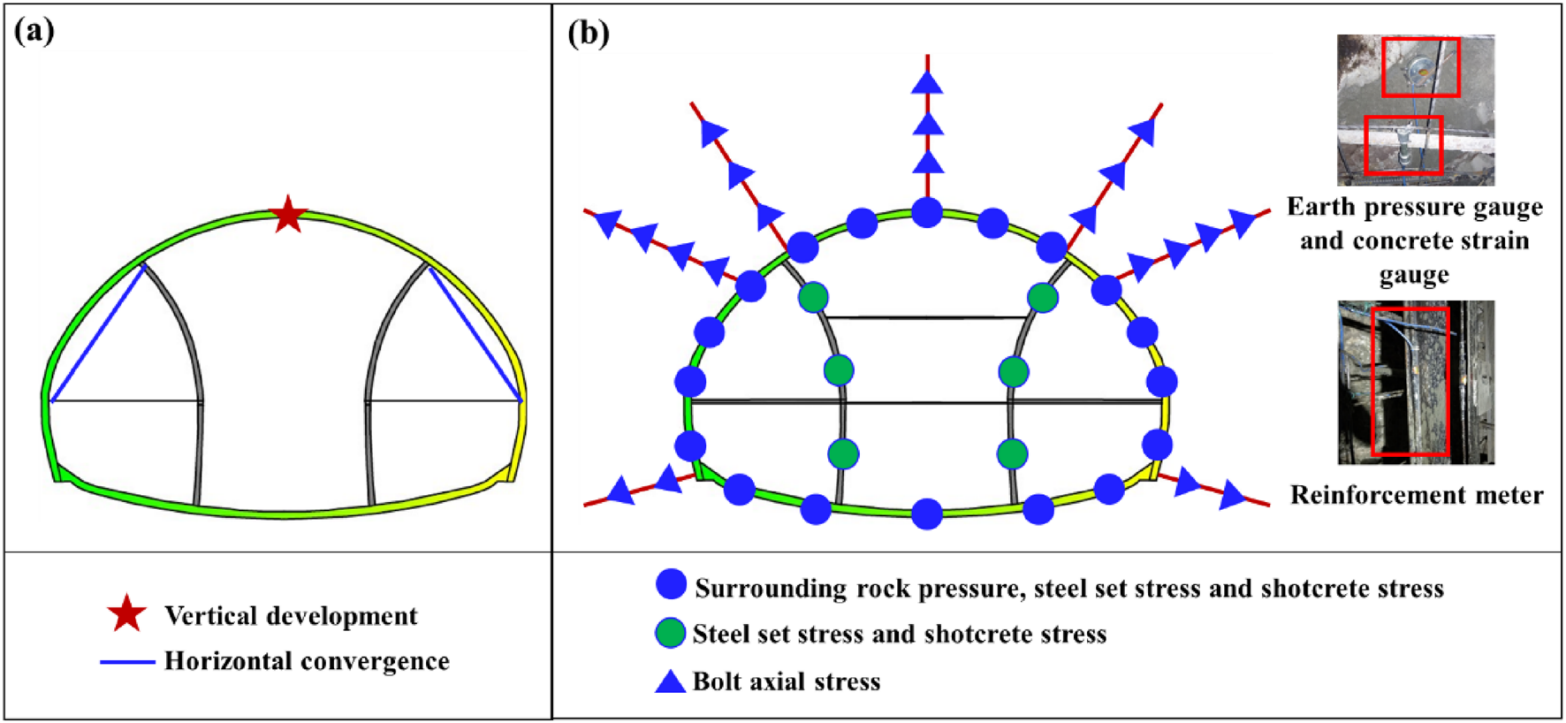
Monitoring scheme: (**a**) Deformation monitoring scheme; (**b**) Stress monitoring scheme.

As shown in Fig. 3, the monitoring schemes include deformation monitoring and mechanical monitoring. The deformation monitoring location is located at section DK215 + 110, where the tunnel is buried at a depth of 31 m, as shown in Fig. 3a, deformation monitoring includes one vertical settlement monitoring point and two horizontal convergence monitoring points for the left and right pilot tunnels. The tunnel mechanical monitoring point is located at DK215 + 105, 5 m away from the deformation monitoring position, as shown in Fig. 3b shows the tunnel mechanical monitoring schemes, including: earth pressure cell, reinforcement stress meter, concrete strain gauge, and bolt axial force meter. Deformation monitoring is very important for tunnel engineering, as it can help engineers detect whether there is excessive deformation in the tunnel, and even predict collapse. For this article, deformation monitoring can help better summarize the deformation characteristics, step construction, and the impact of support structures on deformation of the tunnel. Meanwhile, it can be compared with other tunnels to summarize common patterns. In the stress monitoring project, the earth pressure cell is used to monitor the surrounding rock pressure, which can better explore the load transfer law of this type of tunnel; Reinforcement gauges and concrete strain gauges are used to monitor the stress state of support structures, determine whether they have failed, which can determine whether the tunnel design is reasonable and propose targeted improvements; The bolt axial force meter is used to monitor the stress state of bolts and determine whether they are working properly.

### Analysis of deformation

**Figure 4:**
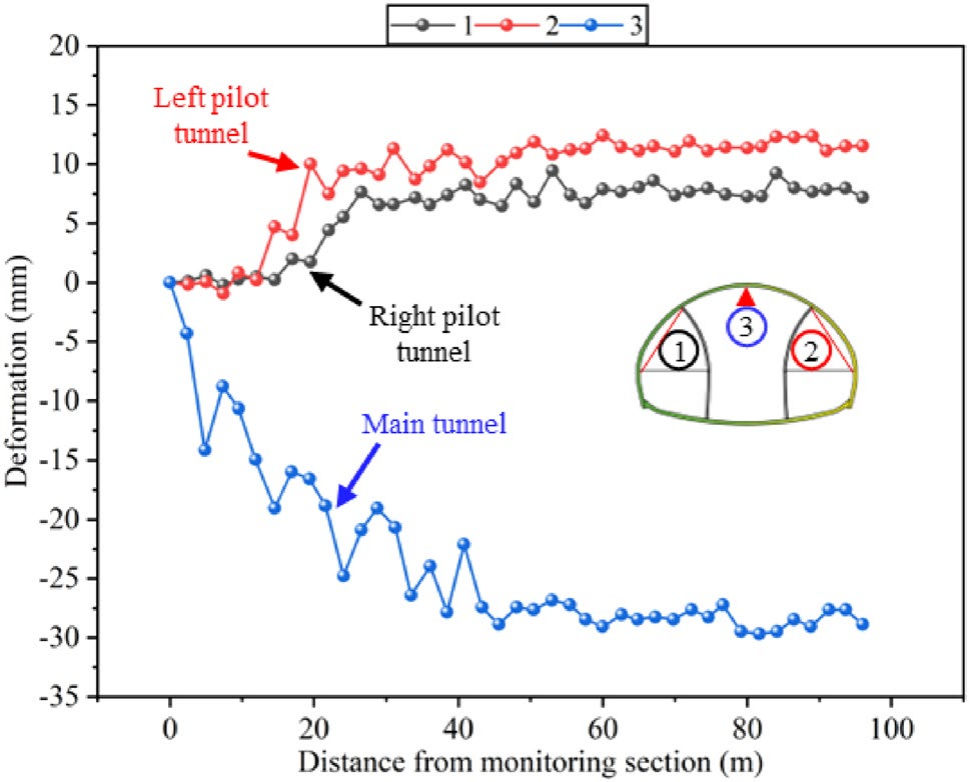
Development curve of tunnel deformation monitoring.

This article stipulates that deformation monitoring results should be positive for deformation inside the tunnel and negative for deformation outside the tunnel. As shown in Fig. 4, the deformation trend of the tunnel first increases rapidly and then tends to stabilize, which is consistent with previous monitoring results. The monitoring of tunnel deformation starts after the excavation of the upper steps of the main tunnel, so the horizontal convergence values of the left and right guide tunnels are more stable and smaller compared to the vertical settlement. The horizontal convergence of the left pilot tunnel is smaller than that of the right pilot tunnel, because the left pilot tunnel is excavated before the right pilot tunnel, resulting in a longer deformation stability time. Due to the small difference in horizontal convergence values, it can be considered that the deformation of the left and right pilot tunnels is symmetrical. The negative horizontal convergence value indicates that the surrounding rocks on both sides are expanding outward from the tunnel. The reason is that the high span ratio of super-large-span tunnels is small, and the loading of arch crown is transmitted to the tunnel walls through steel frame, ultimately leading to outward expansion on both sides of the tunnel. This also indicates that the steel frame and feet-lock bolt combined support structure used in super-large-span tunnels has a good effect on sharing the load on the arch crown and reducing the vertical settlement. Compared with horizontal convergence, the vertical settlement is larger. On the one hand, this is because the monitoring time of vertical settlement is more complete; on the other hand, it indicates that the load borne by the arch is higher than that on both sides of the tunnel. The reason for the significant fluctuations of the vertical settlement is due to the disturbance by step excavation. The three-step excavation of the main tunnel resulted in a higher number of disturbances, but overall, the vertical settlement was within an acceptable range. During the stable deformation period, there is a small fluctuation in deformation due to the temporary support removal.

### Analysis of mechanical

#### Surrounding rock pressure

**Figure 5:**
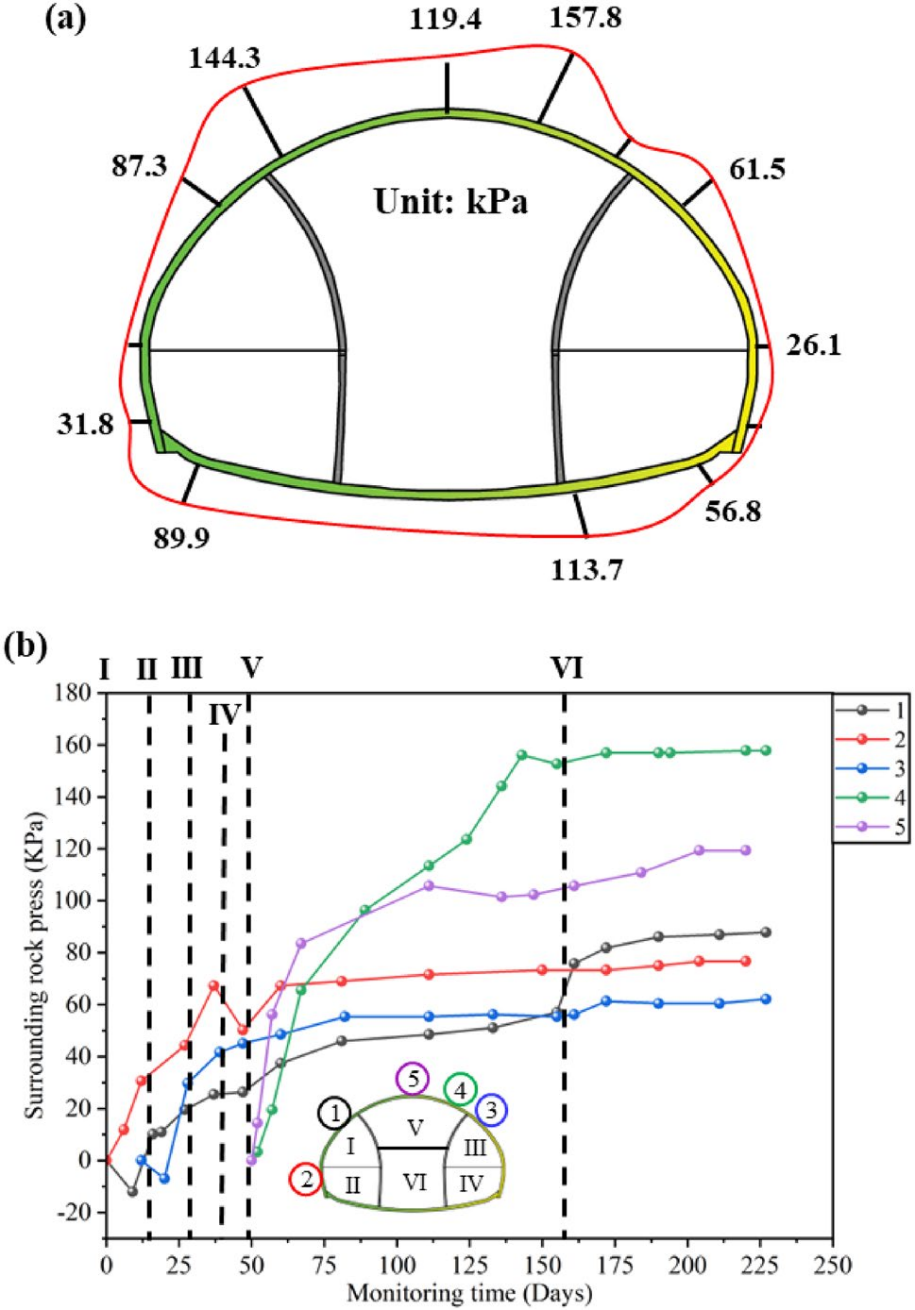
Surrounding rock pressure: (**a**) Distribution of surrounding rock pressure; (**b**) Development curve of surrounding rock pressure.

The mechanical monitoring results in this article are positive for tension and negative for pressure. As shown in Fig. 5a, the maximum value of surrounding rock pressure is 157.8 kPa, located at the right spandrel. From the distribution perspective, the phenomenon of excessive surrounding rock pressure in the first pilot tunnel of Xiabei mountain No. 2 tunnel is relatively mild compared to the monitoring results of Zhao et al.^39^. The main reason is that the temporary support parameters for the Xiabei mountain No. 2 tunnel are more conservative, reducing the transfer of loads from the later pilot tunnel rock mass to the first pilot tunnel, as well as the disturbance caused by step excavation. The temporary support of the Qichong village tunnel is made of 15 cm thick C25 shotcrete, with the same steel frame and steel mesh model as the Xiabei mountain No. 2 tunnel. The overall strength of the support structure is lower than that of the Xiabei mountain No. 2 tunnel. Moreover, during the temporary support of the Xiabei mountain No. 2 tunnel, bolts will be applied in the direction of the main tunnel, greatly enhancing the mechanical performance of the temporary support. Compared with the Qichong village tunnel, in addition to the higher strength of the temporary support structure, the burial depth of the Xiabei mountain No. 2 tunnel is deeper. From this, it can be seen that calculating the overburden weight alone is not enough to determine the surrounding rock pressure of this type of tunnel. The calculation method for the surrounding rock pressure of super-large-section tunnels using the step excavation method still needs further discussion.

As shown in Fig. 5b, the development trend of surrounding rock pressure shows a rapid increase and then a gradually stable trend, which is similar to the monitoring results of Luo et al.^7^, Zhou et al.^39^, Zhao et al.^5^ and others. The fluctuation section in the middle of the time history curve is mainly caused by step excavation. Among them, the excavation of the upper steps of the main tunnel has a significant impact on the surrounding rock pressure of the vault, and after the excavation of the upper steps of the main tunnel, the surrounding rock pressure of the vault has increased by more than twice. It can be seen that even if the temporary support strength is high, the surrounding rock pressure of the super-large-section tunnel excavated in stages is still affected by the step excavation, and it will have a significant impact.

#### Shotcrete axial force

**Figure 6:**
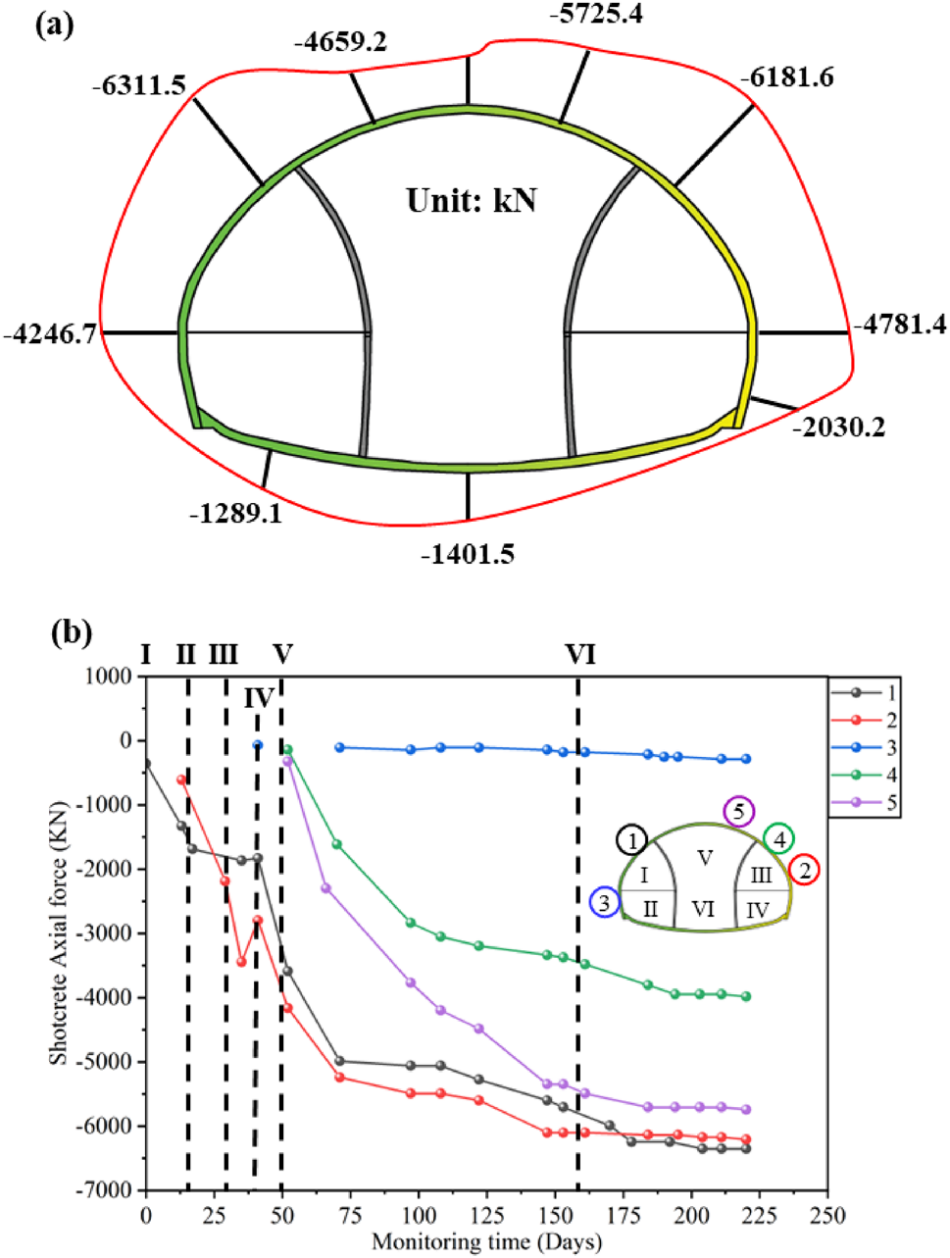
Shotcrete axial force monitoring: (**a**) Distribution of shotcrete axial stress; (**b**) Development curve of shotcrete axial stress.

The axial force distribution of the primary support shotcrete is shown in Fig. 6a. The shotcrete is in a compressed state as a whole, with the maximum axial force located at the left spandrel; The minimum axial force is located on the left of inverted arch. The overall axial force of the lower step is smaller than that of the upper step. Based on the deformation monitoring results, it can be concluded that the combined support system composed of steel frame and feet-lock bolt has a good effect on limiting deformation and bearing capacity of the upper step.

Figure 6b shows the time history curve of the axial force of the primary support shotcrete. The excavation of main tunnel has a significant impact on the shotcrete axial force. The excavation of main tunnel increases the axial force of the left and right arch waists by about − 3540 kN and − 3160 kN, respectively. After the excavation of the middle and lower steps of the main tunnel, except for the lower axial force on the right side reaching 1068 kN, which exceeded the tensile strength of the concrete, the measured values at all other measuring points showed a sudden upward trend. It can be seen that step excavation has a significant impact on the axial force of other pilot tunnels.

#### Steel frame axial force and bending moment

**Figure 7:**
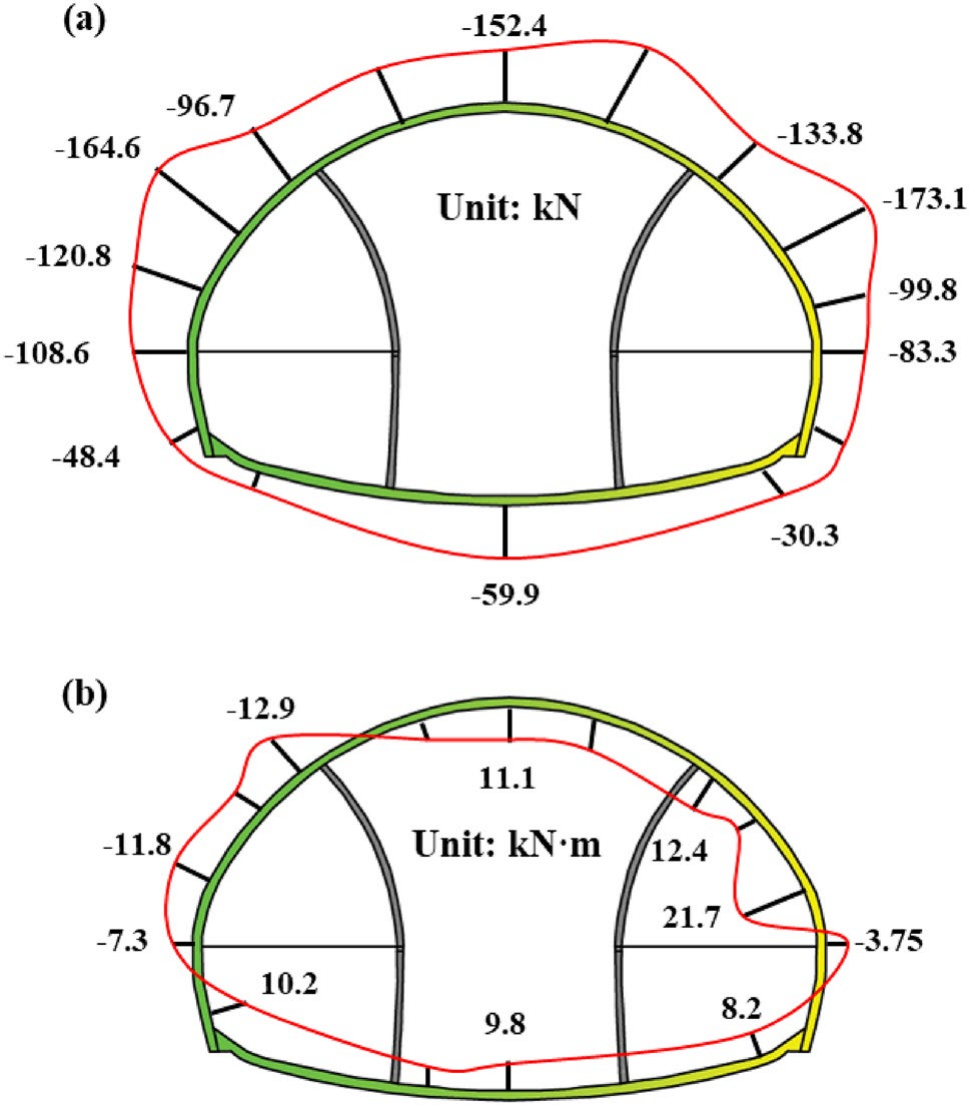
Steel frame monitoring: (**a**) Distribution of steel frame axial force; (**b**) Distribution of steel frame bending moment.

The mechanical distribution of the steel frame is shown in Fig. 7a and b. The mechanical distribution of steel frame also has the characteristics that the upper structure is greater than the lower structure. The maximum axial force is located on the left and right arch waists, and the maximum bending moment is also located on the left and right arche waists. It can be seen that the arch waist undergoes significant deformation towards the tunnel outside because of the vault load, and the support here should be strengthened.

**Figure 8:**
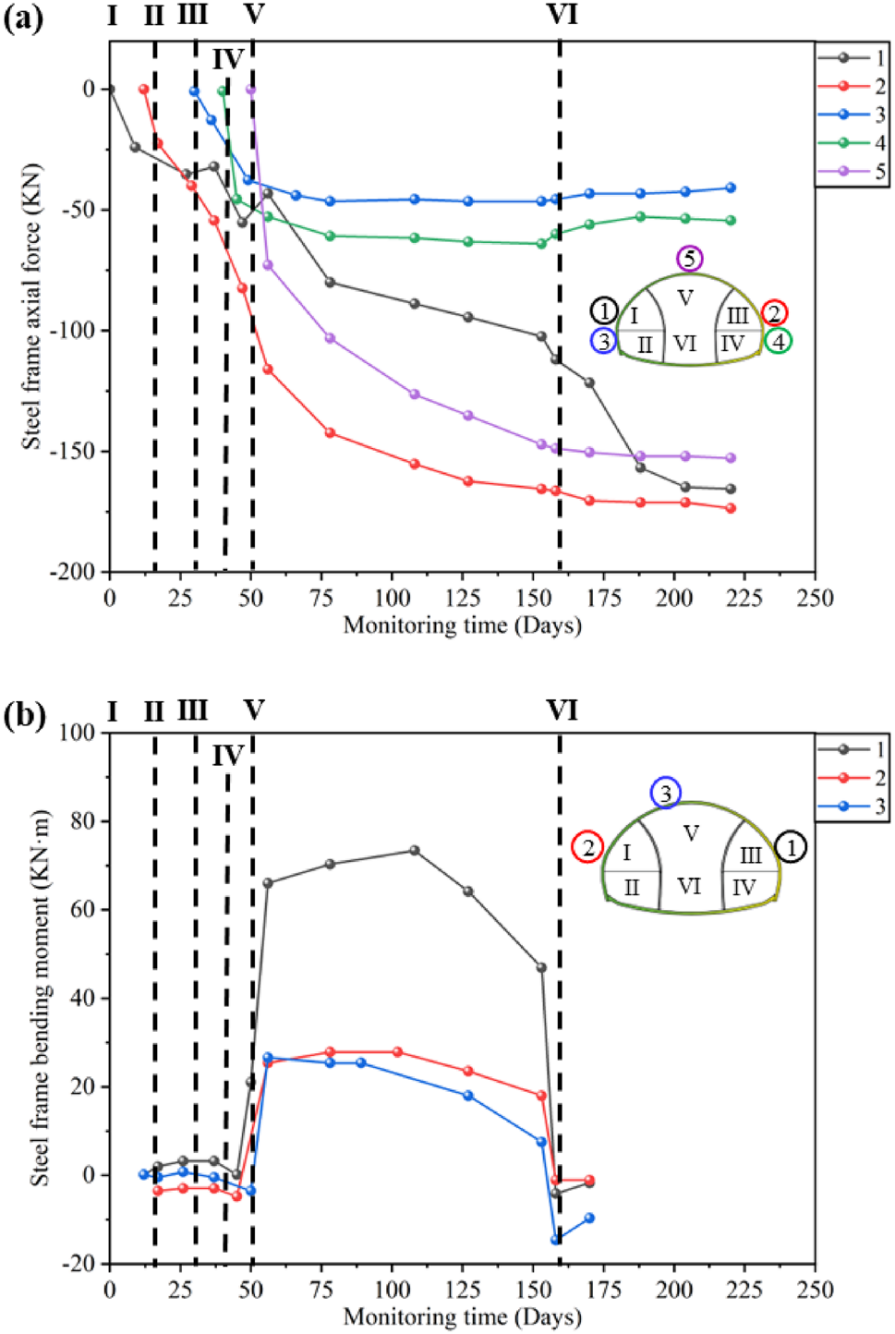
Steel frame monitoring: (**a**) Development curve of steel frame axial force; (**b**) Development curve of steel frame bending moment.

From Fig. 8a, It can be seen that after the excavation of the upper steps of the left and right pilot tunnels, the axial force on the steel frame rapidly increases to − 35 to − 30 kN, and then tends to stabilize until before the excavation of the upper steps of the main tunnel. After the excavation of the upper steps on the main tunnel, the axial force on the left and right arch waists increased by about − 100 to − 60 kN, which was more than twice as much as before. It can be seen that the excavation of the upper step on the main tunnel has a significant impact on the support structures of the left and right pilot tunnels. After the main tunnel is excavated, the stress on both sides of the arch waist significantly increases, indicating that it is necessary to enhance temporary support to assist in bearing the vault load before excavating the main tunnel.

#### Bolts axial force

**Figure 9:**
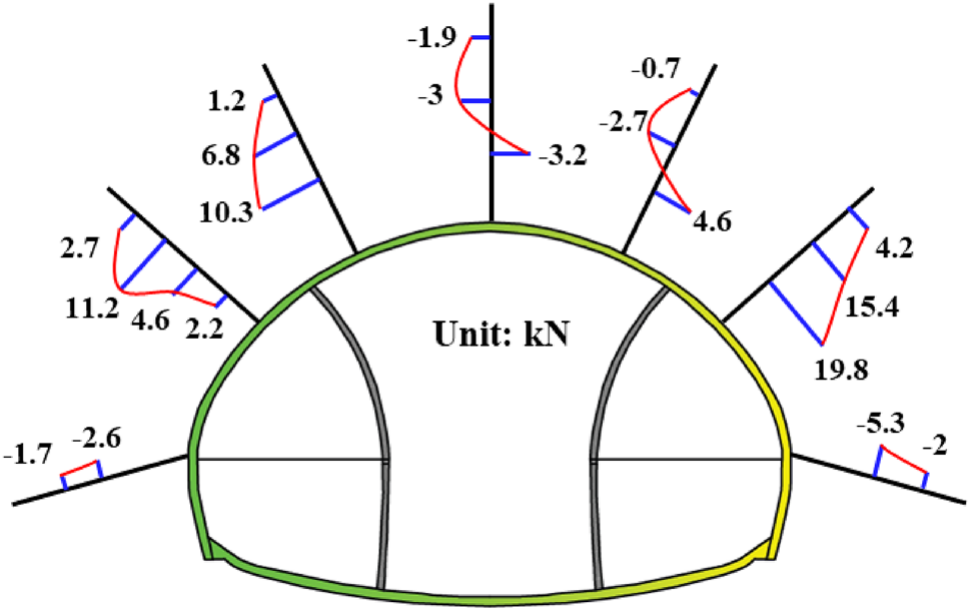
Distribution of bolts axial force.

To analyze the effect of bolts, draw the axial force distribution of bolts on the monitoring section as shown in Fig. 9. The tunnel adopts self-advancing bolts to reinforce the surrounding rock, and the mechanism of this type of bolt reinforcement is provided by the frictional force generated by the adhesive, surrounding rock, and bolt. Although the bolt axial force meter used in the study did not have a self-advance function, the same adhesive was injected during monitoring, so the bolt axial force meter can better reflect the actual reinforcement effect. It can be seen that after the tunnel excavation is completed, the bolts at the vault do not have a significant tensile effect, this is similar to previous research results^39^. The bolts on both sides of the tunnel have a good effect on suppressing the deformation of the surrounding rock and should not be cancelled. For super-large-section tunnels, the role of the feet-lock bolt is not only to anchor the rock, but more importantly, to combine with the steel frame, thereby limiting the displacement of the steel frame, and avoid significant vertical settlement. As shown in Fig. 9, the feet-lock bolt is under compression, indicating that the feet-lock bolt has not played a role in anchoring the rock mass, which is inconsistent with existing monitoring results. In order to combing with steel frame, the feet-lock bolt needs to be welded to the steel frame. When the surrounding rock is relatively loose, the load on the vault is transmitted to both sides, causing the feet-lock bolt and steel frame to compress the surrounding rock on both sides together, generating pressure. Although the feet-lock bolt did not have the effect of anchoring the rock mass, combined with deformation, surrounding rock pressure, and steel frame axial force monitoring results, it can be seen that feet-lock bolt plays a significant role in limiting the vertical settlement and sharing the surrounding rock pressure. Overall, the bolts on the vault has almost no effect on reinforcing the rock mass and supporting the bearing capacity of the supporting structure, which is similar to the research results of Zhao et al.^39^. It can be seen that the bolts on the vault for shallow-buried super-large-section tunnels can be cancelled and replaced by stronger shotcrete, steel frames, or temporary supporting structures.

#### Temporary support structures axial force and bending moment

**Figure 10:**
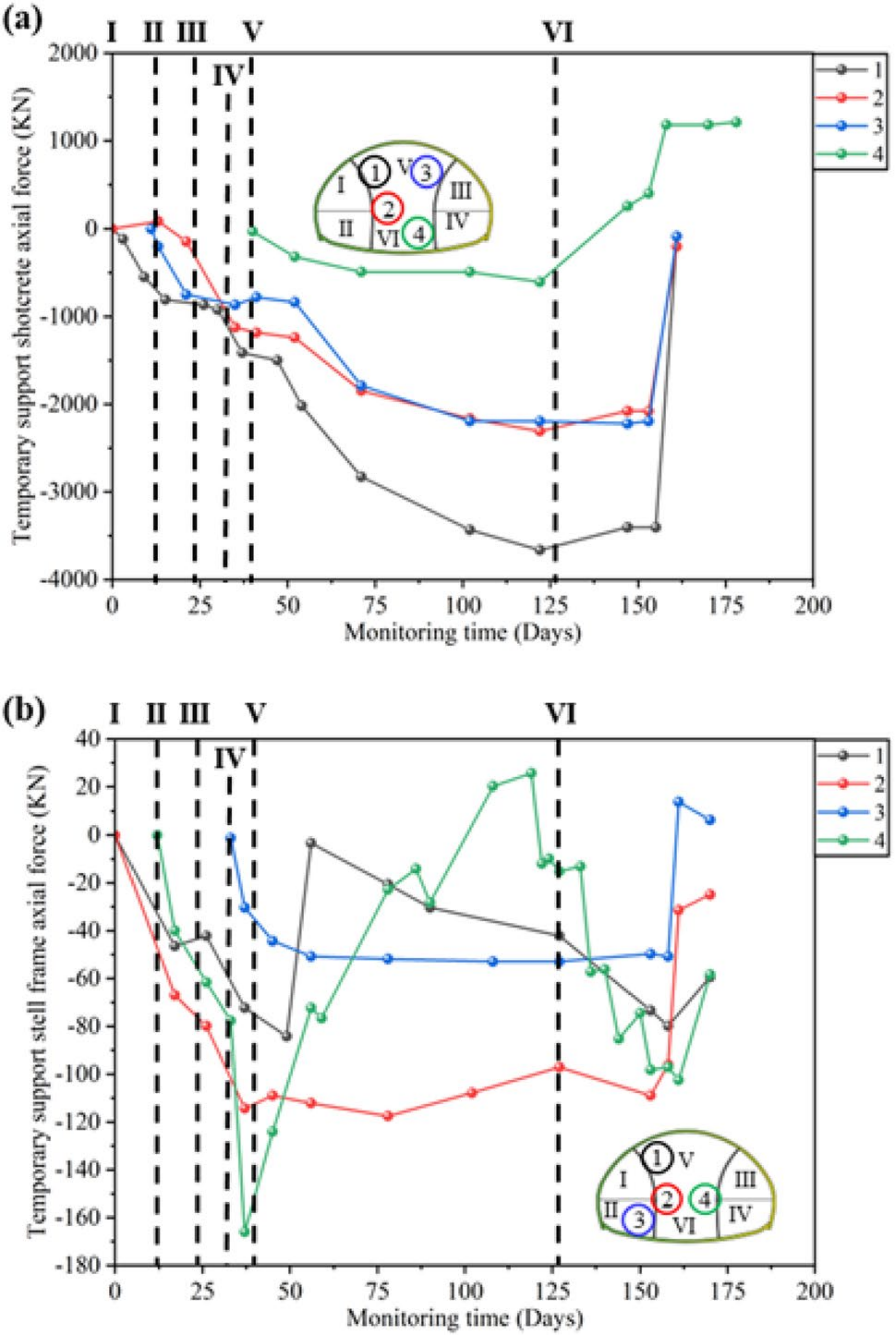
Temporary support monitoring: (**a**) Development curve of temporary support shotcrete bending moment; (**b**) Development curve of temporary support shotcrete axial force.

The axial force and bending moment monitoring curve of the temporary support steel frame is shown in Fig. 10. The excavation of the lower steps of the left pilot tunnel and the upper steps of the main tunnel has a significant impact on the axial force of the steel frame. After the excavation of the middle and lower steps of the main tunnel, the monitoring points for axial force 1, 2, and 3, as well as bending moment 2, and 3, almost decreased to 0. The temporary support has a significant limiting effect on the soil of the main tunnel, indicating the necessity of temporary support. The step excavation has caused frequent adjustment of the force on the temporary support steel frame. After the excavation of the middle and lower steps of the main tunnel, the support force provided by the steel frame has significantly decreased. This indicates that the temporary support structure has a very significant limiting effect on the pressure of the vault surrounding rock. Combined with Figs. 5, 6, 7 and 8, it can be seen that from the removal of temporary support to the construction of secondary lining, the primary support stress increases again, indicating that the removal of temporary support will lead to stress redistribution again. Therefore, when dismantling temporary support structures, emphasis should be placed on monitoring displacement and stress changes.

### Analysis of safety factor of support structure

The safety factor calculation of shotcrete in the TB 10003-2016. 2016. Code for design of railway tunnel.^38^ can effectively quantify the safety of support structures. According to the monitoring results, the minimum safety factor of shotcrete at each step of the monitoring section during the construction process and the axial force and bending moment at its position are calculated, as shown in Tables 2 and 3. When calculating, the compressive strength of concrete is taken as 20.1 MPa, and the tensile strength is taken as 2.01 MPa.

**Table 2:** Minimum safety factor for primary support shotcrete.

Construction step	Least safe location	Safety factor	Maximum bending moment (kN m)	Maximum axial force (kN)
3	I	7.63	122.1	− 1637.2
2	II	2.32	189.4	− 3469.1
4	III	3.93	171.4	− 3182.2
5	IV	2.39	234.7	− 5217.5
7	I	2.01	239.9	− 6210.7
Inverted arch	I	1.98	272.6	− 6311.5

**Table 3:** Minimum safety factor for temporary support shotcrete.

Construction step	Least safe location	Safety factor	Maximum bending moment (kN m)	Maximum axial force (kN)
3	I	6.21	47.4	− 1275.2
2	I	4.72	79.6	− 1677.4
4	II	2.14	186.5	− 1432.6
5	III	1.56	315.8	− 2951.4

According to Tables 2 and 3, the minimum safety factor and its location of the shotcrete on the monitoring section are constantly changing with the construction process. The position with the lowest safety factor mostly occurs in the left pilot tunnel, especially the upper steps, which once again confirms the phenomenon that the first pilot tunnel is affected by the excavation of the subsequent pilot tunnels. Excavation also has a significant impact on adjacent support structures. For primary support, excavation of the main tunnel and construction of the inverted arch have the greatest disturbance; for temporary support, the excavation of the main tunnel causes the greatest disturbance.

Overall, the primary support of Xiabei mountain No. 2 tunnel fully meets the safety requirements, and the safety factor of the left pilot tunnel has been significantly reduced due to multiple disturbances. Therefore, for this type of tunnel, the primary and temporary support of the first pilot tunnel can be enhanced, while the primary and temporary support of other parts can be appropriately reduced. The existing calculation formula for surrounding rock pressure cannot reflect the above issues, and its effectiveness in guiding the design and construction of such tunnels is limited.

## Calculation formula for surrounding rock pressure considering step excavation

### Overview

Based on Section 3 analysis of the support mechanical response of the Xiabei mountain No. 2 tunnel, it was found that the surrounding rock pressure of the super-large-section tunnel is influenced by many factors, including:

1. The limitation of temporary support structures on surrounding rock pressure;
2. The influence of spatial effects on each pilot tunnel during segmented excavation;
3. Deterioration effect of surrounding rock in post excavation pilot tunnel.

Obviously, it is difficult to consider all the above factors through theoretical formulas, but in fact, the reason for the change in surrounding rock pressure can be attributed to step excavation. According to the analysis results in Section 3, it can be concluded that the first pilot tunnel is affected by the disturbance of the later pilot tunnel, and the surrounding rock pressure will continue to rise. Luo et al. constructed a surrounding rock pressure calculation method considering the mutual influence of left and right pilot tunnels based on surrounding rock pressure monitoring data, and achieved good application results^36,37^. The reasonable construction of an empirical model for surrounding rock pressure based on monitoring data has a good application effect on super-large-section tunnels with complex construction mechanical responses. In addition to section DK215 + 105, this article also monitored the surrounding rock pressure of the other four sections of Xiabei mountain No. 2 tunnel and five sections of Xiabei mountain No. 1 tunnel. Xiabei mountain No. 1 tunnel is only more than 200 m away from Xiabei mountain No. 2 tunnel, and the tunnel lithology is similar to that of tunnel sections, all of which are shallow buried tunnels. Based on sufficient monitoring data, an empirical formula for surrounding rock pressure considering step excavation is constructed.

### Calculation formula for surrounding rock pressure

**Figure 11:**
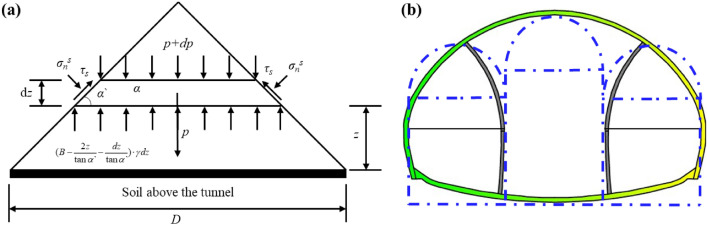
Calculation model for surrounding rock pressure: (**a**) Single hole calculation model; (**b**) Division of super-large-span tunnel pilot tunnels.

Equations (1)–(5) are the calculation formulas for the surrounding rock pressure of shallow buried tunnels derived by Wang based on the Terzaghi principle^44^. This formula effectively predicts the surrounding rock pressure of normal shallow buried tunnels. The Xiabei mountain No. 2 Tunnel studied in this article is a super large cross-section tunnel that adopts step excavation method. The step excavation method causes the surrounding rock of the first pilot tunnel to be continuously disturbed, and the surrounding rock pressure continues to develop. Therefore, Wang's formula is not applicable to this type of tunnel. In order to fully consider the impact of step excavation, this paper proposes the influence coefficient of step excavation pilot tunnels based on sufficient on-site monitoring data, and based on this, an empirical formula for surrounding rock pressure considering step excavation was proposed. The specific calculation steps are as follows: Firstly, simplify the tunnel excavated by the double-side-wall pilot tunnel method into three straight wall arched tunnels. Then, through Eq. (1) calculate the surrounding rock pressure of three pilot tunnels without the influence of step excavation. Calculate the empirical parameters of step excavation based on measured surrounding rock pressure data. The final rock pressure value can be obtained by combining the surrounding rock pressure without the influence of step excavation with the step excavation empirical parameters. At this point, the surrounding rock pressure of the first pilot tunnel is Eq. (6), the surrounding rock pressure of the second excavation of the pilot tunnel is Eq. (7), the final pilot tunnel was not disturbed subsequently, so it was directly obtained. The final surrounding rock pressure of the tunnel is the highest surrounding rock pressure among the three pilot tunnels (Eq. (8)). The specific calculation model is shown in Fig. 11.

1 $$p = \frac{{\frac{\gamma \tan \alpha^{\prime}}{2}}}{{1 - \frac{m\tan \alpha ^{\prime}}{2}}} \cdot D$$

2 $$m = - \frac{2}{\tan \alpha ^{\prime}} + \frac{{4(\sin^{2} \theta_{1H}^{s} + K_{a} \cos^{2} \theta_{1H}^{s} )\cos^{2} \varphi \cos \alpha ^{\prime}}}{{\left[ {1 + K_{a} - \frac{{(1 - K_{a} )\sin \theta_{1H}^{s} \cos \theta_{1H}^{s} }}{{\theta_{1H}^{s} }}} \right] \cdot [1 - \sin (2\alpha ^{\prime} + \varphi )\sin \varphi ]}}$$

3 $$\theta_{1H}^{s} = \frac{{\arctan \left( {\frac{4\lambda }{{1 + 4\lambda^{2} }}} \right)}}{\pi /4}\varphi + \frac{\pi }{4} - \frac{\varphi }{2}$$

4 $$\alpha ^{\prime} = \frac{{\arctan \left( {\frac{4\lambda }{{1 + 4\lambda^{2} }}} \right)}}{\pi /4}\varphi + \frac{\pi }{2} - \varphi$$

5 $$\lambda = \frac{\Delta u}{D}$$

where *γ* is the density of soil, *α*^′^ is angle between the shear plane and the horizontal direction, *m* is a parameter related to *φ*(soil strength), *θ*^s^_1H_ (principal stress rotation angle) and *α*^′^(shear plane rotation angle).

6 $$q_{1} ^{\prime} = k_{1} q_{2} + k_{2} q_{3} + q_{1}$$

7 $$q_{2} ^{\prime} = k_{3} q_{3} + q_{2}$$

where *k*_1_, *k*_2_, *k*_3_ are the step excavation empirical parameters, determined by the measured changes in surrounding rock pressure from multiple monitoring sections; *q*_1_, *q*_2_, *q*_3_ are the initial rock pressures of the first pilot tunnel, the second pilot tunnel, and the main tunnel, respectively, determined by Eq. (1); *q*_1_^′^ and *q*_2_^′^ respectively represent the surrounding rock pressure of the first and second pilot tunnels affected by step excavation.

8 $$q_{ultra} = \max \left( {q_{1} ^{\prime},q_{2} ^{\prime},q_{3} } \right)$$

where *q*_*ultra*_ is the final surrounding rock pressure value.

### The step excavation empirical parameters

**Figure 12:**
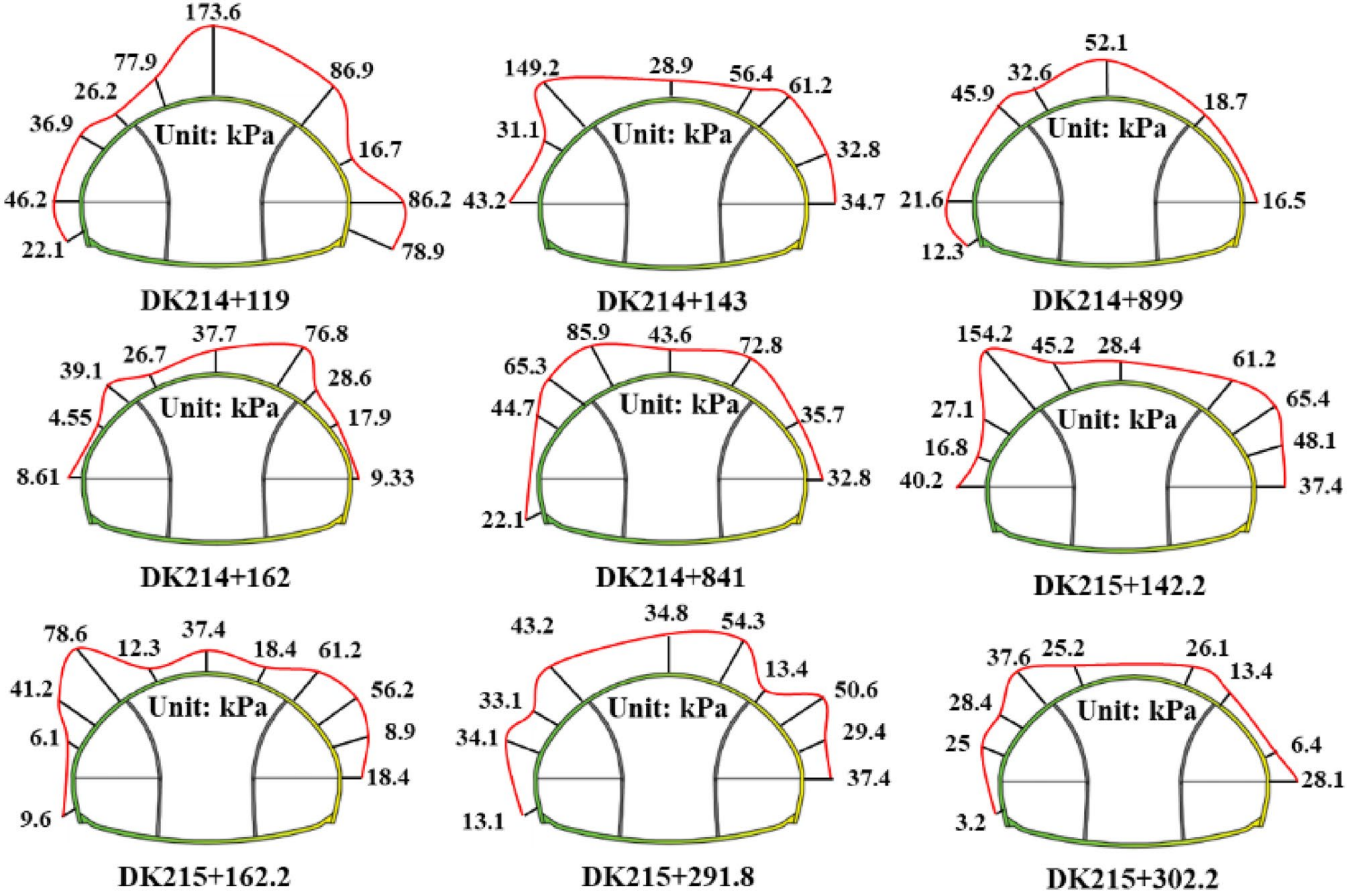
Distribution of surrounding rock pressure in other monitoring sections.

The distribution of surrounding rock pressure in other monitoring sections is shown in Fig. 12. The main steps for calculating the step excavation empirical parameters are as follows:

1. Statistics the maximum surrounding rock pressure values at different pilot tunnel of each monitoring section;
2. Based on the monitoring curves of the maximum surrounding rock pressure values of each pilot tunnel, determine the step excavation empirical parameters of each pilot tunnel.

Table 4 shows the specific calculation process of step excavation empirical parameters.

**Table 4:** Determination of empirical parameters considering step excavation.

	a	b	c			a	b	c	
Section 1					Section 2				
1	20	4	k1	0.0650	1	27	5	k1	0.1082
2	24				2	32			
3	87.3	63.3	k2	0.4011	3	86.9	54.9	k2	0.3162
4	43				4	27			
5	61.5	18.5	k3	0.1172	5	46.2	19.2	k3	0.1106
6	157.8				6	173.6			
Section 3					Section 4				
1	19	5	k1	0.0817	1	10	2	k1	0.1070
2	24				2	12			
3	149.2	125.2	k2	2.2199	3	45.9	33.9	k2	0.6507
4	43				4	6			
5	61.2	18.2	k3	0.3227	5	18.7	12.7	k3	0.2438
6	56.4				6	52.1			
Section 5					Section 6				
1	4	3	k1	0.0840	1	12	3	k1	0.0840
2	7				2	15			
3	39.1	32.1	k2	0.5856	3	65.3	50.3	k2	0.5856
4	11				4	27			
5	28.6	17.6	k3	0.1013	5	35.7	8.7	k3	0.1013
6	76.8				6	85.9			
Section 7					Section 8				
1	14.2	2.2	k1	0.0564	1	8	3	k1	0.0593
2	16.4				2	11			
3	65.4	49	k2	0.3178	3	43.2	32.2	k2	0.5930
4	17				4	43			
5	39	22	k3	0.1427	5	50.6	7.6	k3	0.1340
6	154.2				6	54.3			
Section 9					Section 10				
1	24	3.8	k1	0.0982	1	14	2.8	k1	0.0996
2	27.8				2	16.8			
3	58.7	30.9	k2	0.3931	3	37.6	20.8	k2	0.7969
4	18.7				4	23			
5	38.5	20	k3	0.2544	5	28.1	5.1	k3	0.1954
6	78.6				6	26.1			

“1” is the surrounding rock pressure of the first pilot tunnel when the excavation of the second pilot tunnel begins; “2” is the surrounding rock pressure of the first pilot tunnel after the excavation of the second pilot tunnel is completed; “3” is the final surrounding rock pressure of the first pilot tunnel; “4” is the surrounding rock pressure of the second pilot tunnel when the main tunnel starts excavation; “5” is the final surrounding rock pressure of the second pilot tunnel; “6” is the surrounding rock pressure of the main tunnel.

“a” is on-site monitoring surrounding rock pressure value; “b” is changes in surrounding rock pressure; “c” is step excavation empirical parameters.

The result of monitoring Section 3 shows a significant deviation compared to other sections, due to the presence of a slip layer above the left pilot tunnel, which has a significant impact on the universality of the results. According to the Shoveler principle, it should be excluded. In summary, based on the average value of the monitoring data mentioned above, the step excavation empirical parameters can be obtained as *k*_1_ = 0.0845, *k*_2_ = 0.4777, *k*_3_ = 0.1613.

### Verification

The specific verification steps for section DK215 + 105 are as follows:

1. As shown in Fig. 12, the section DK215 + 105 of Xiabei mountain No. 2 tunnel is simplified into a mechanical model consisting of three straight wall arched tunnels.
2. Using Eq. (1) calculate the surrounding rock pressure of three straight wall arch tunnels to obtain *q*_1_, *q*_2_ and *q*_3_.
3. Substitute *q*_1_, *q*_2_ and *q*_3_ into Eqs. (6)–(7) obtain *q*_1_^′^ and *q*_2_^′^.
4. Using Eq. (8) obtain the maximum surrounding rock value of the monitoring section.

As shown in Table 5, the calculation results indicate that the proposed theoretical calculation model for surrounding rock pressure is more accurate, and can also calculate the surrounding rock pressure of each of the three pilot tunnels in step excavation, providing a good reference for tunnel engineering using segmented excavation.

**Table 5:** Calculation results and comparison of surrounding rock pressure.

Calculation methods	Calculation results (kPa)			Parameter description
	First	Second	Main	
RMR method	494.5			*S*_RMR_ = 26, *B* = 26m, *γ* = 25.7 kN m^−3^
Terzaghi method	580.1–796.7			The height of the soil cover is 31 m, and the height of the stratum arch *H*_pt_ = 22.6–31 m
Proposed method	133.0	82.5	143.8	–
Monitoring values	87.3	61.5	157.8	–

## Discussion

The research presented in this article indicates that the mechanical response and surrounding rock pressure of shallow buried super large span tunnels constructed using the step excavation method will continuously change with the progress of construction steps. The previous model experiments, numerical simulations, and on-site monitoring results have limited guidance for this type of tunnel. The traditional formula for calculating the surrounding rock pressure neglects the influence of step excavation on the surrounding rock pressure. The analysis conducted to explore the construction mechanical response and rock pressure evolution law of shallow buried super large span tunnels in weak surrounding rock includes: (1) Carry out deformation and stress monitoring at Xiabei mountain tunnel; (2) Analyze and summarize the deformation, support structure stress law, and surrounding rock pressure transmission law of this type of tunnel; (3) Based on the monitoring data of surrounding rock pressure from 10 monitoring sections, the empirical coefficient of step excavation was obtained, and the calculation formula for surrounding rock pressure considering step excavation was derived.

Due to the lack of similar projects to refer to in the past, the support design of the Xiabei mountain tunnel is relatively conservative, resulting in material waste or insufficient support for local support. According to the monitoring results of the bolt axial force meter (Fig. 9) and previous research results, it is known that the effect of the bolts of tunnel arch on shallow buried large-span tunnels is limited, and it is recommended to cancel^39^. The joint support system formed by feet-lock bolt and steel frame has a significant optimization effect on the load transfer of this type of super large span tunnel. The monitoring results showed that the feet-lock bolt did not play a role in reinforcing the rock mass. Therefore, a more effective anchoring agent should be used to strengthen the bonding force between the feet-lock bolt and the surrounding rock, thereby further improving the ability of the feet-lock bolt to reinforce the rock mass. The disturbance caused by excavation of the later pilot tunnel can be reduced by removing the temporary support of the first pilot tunnel before excavation, in addition to strengthening the support. For grade III surrounding rock, the current primary support strength is too high, resulting in material waste. Therefore, the thickness of the primary support can be appropriately reduced. On this basis, it is recommended that the primary support strength of the first pilot tunnel be slightly higher than that of the later pilot tunnel, in order to reduce the impact of excavation disturbance on the later pilot tunnel. The temporary support strength is also too high, it is recommended to reduce it. For grade V surrounding rock, the primary support strength of the later pilot tunnel can be reduced, and the temporary support on the side wall of the first pilot tunnel can be appropriately reduced.

## Conclusion

Based on the measured monitoring data of Xiabei mountain No. 1 and No. 2 tunnel, the mechanical response of the support structure of a super-large-section tunnel with weak surrounding rock was analyzed, and a calculation formula for the surrounding rock pressure of a shallow-buried super-large-section tunnel suitable for the use of step excavation method was derived. The main conclusions obtained from the study are as follows:

1. When excavating shallow-buried super-large-section tunnels in weak strata using the double-side-wall pilot tunnel method, it is crucial to manage the deformation and stress of the primary tunnel support effectively. This process involves rapid development, stability, and fluctuations during step excavation and temporary support removal. To address this, the utilization of feet-locking bolts and steel frame welding can distribute pressure on the surrounding rock and limit vertical settlement. Special attention should be given to deformation and stress during construction phases and temporary support removal. Actively incorporating feet-locking bolts is recommended to optimize primary support stress.
2. Utilizing high-strength temporary support and applying bolts to unexcavated pilot tunnel soil can minimize the impact of subsequent tunnel excavation on the initial pilot tunnel. Temporary support complements the steel frame in withstanding vault rock pressure. The excavation of main tunnel upper steps significantly influences the surrounding rock pressure of other pilot tunnels, making it challenging to calculate accurately using existing methods.
3. The primary safety concern typically arises in the left pilot tunnel, especially on its upper steps. Main tunnel excavation and inverted arch construction pose the highest disturbance to primary support, while main tunnel excavation affects temporary support the most. Enhancing primary and temporary support for the first pilot tunnel is essential due to multiple disturbances. For shallow-buried super-large-section tunnels utilizing step excavation, reinforcing support for the initial pilot tunnel while appropriately reducing support for other sections is recommended.
4. An empirical parameter derived from rock pressure data of the Xiabei mountain No. 1 and No. 2 tunnels was used to develop a calculation formula for surrounding rock pressure considering step excavation. This formula offers more accurate calculations for super-large-section tunnels and its individual pilot tunnels, proving valuable for similar tunnel projects in weak surrounding rock with step excavation methods.

## Data Availability

All the required data for the research has been reflected in Manuscript. Specifically, as shown in Figs. 1, 2, 4, 5, 6, 7, 8, 9, 10 and 12; Tables 1, 2, 3, 4, 5.
